# Patient-Derived Organoids in Precision Medicine: Drug Screening, Organoid-on-a-Chip and Living Organoid Biobank

**DOI:** 10.3389/fonc.2021.762184

**Published:** 2021-12-30

**Authors:** Zilong Zhou, Lele Cong, Xianling Cong

**Affiliations:** ^1^ Biobank, China-Japan Union Hospital of Jilin University, Changchun, China; ^2^ Department of Dermatology, China-Japan Union Hospital of Jilin University, Changchun, China

**Keywords:** organoids, patient-derived organoid, living biobanks, microfluidics, drug screening, organoids-on-a-chip

## Abstract

Organoids are *in vitro* self-assembling, organ-like, three-dimensional cellular structures that stably retain key characteristics of the respective organs. Organoids can be generated from healthy or pathological tissues derived from patients. Cancer organoid culture platforms have several advantages, including conservation of the cellular composition that captures the heterogeneity and pharmacotypic signatures of the parental tumor. This platform has provided new opportunities to fill the gap between cancer research and clinical outcomes. Clinical trials have been performed using patient-derived organoids (PDO) as a tool for personalized medical decisions to predict patients’ responses to therapeutic regimens and potentially improve treatment outcomes. Living organoid biobanks encompassing several cancer types have been established, providing a representative collection of well-characterized models that will facilitate drug development. In this review, we highlight recent developments in the generation of organoid cultures and PDO biobanks, in preclinical drug discovery, and methods to design a functional organoid-on-a-chip combined with microfluidic. In addition, we discuss the advantages as well as limitations of human organoids in patient-specific therapy and highlight possible future directions.

## Introduction

Cancer is a heterogeneous disease that includes a complex ecosystem of diverse cell types. Apart from neoplastic cells, tumors include cancer-associated stromal cells, growth factors and metabolites in the microenvironment, which have profound effects on tumor cell growth, invasion ability, and drug response (1). Therefore, these microenvironmental elements are critical in the development of pathologically relevant culture models to study cancer progression. For decades, preclinical cancer research has relied on cell lines as *in vitro* representations of tumor heterogeneity. Traditional drug development is carried out *via* two-dimensional (2D) tumor cell line cultures and transplantation of patient-derived tumor xenografts in animals (2). However, there are several drawbacks in these approaches. For instance, 2D cell line cultures poorly reflect the native microenvironment of tumor tissue, and after many passages in culture, cancer cell lines lose the genetic heterogeneity of parental tumors because of clonal selection (3, 4). This contributes to the low success rate of newly developed drugs in clinical trials (5, 6). Organoids are self-organizing, three-dimensional (3D) structures that are grown *in vitro* from stem cells, and resemble the organ from which the cells were derived (2, 7). The starting cells could be adult stem cells, cancer stem cell, or cancer tissue-derived spheroids. Organoids preserve many structural and functional features such as cell composition and tissue architecture of their corresponding *in vivo* organs.

Clevers et al. developed crypt-villus organoids from a single Lgr5^+^ stem cell using the WENR (Wnt3a+EGF+Noggin+R-spondin-1) protocol that allowed long-term culture and differentiation of primary epithelial cells isolated from intestinal tissue (7–9). The key components of the culture medium included the ligand of LGR5 R-spondin-1, the Wnt pathway agonist, epidermal growth factor (EGF), and bone morphogenetic protein pathway inhibitor Noggin. In addition, the anoikis antagonist Rho-kinase inhibitor Y-27632 is a key factor for improving the success rate of organoid culture. The genotype and genetics of organoids derived from adult stem cells are consistent with those of their parental tissues and remain stable for a long time (10, 11). Another strategy involving the use of pluripotent stem cells (PSCs) has been applied to generate organoids resembling the brain (12), intestine (13), kidney (14, 15) and retina (16, 17).

From 2009 to 2021, use of organoid technology has been rapidly increasing in cancer research, especially for therapeutic screening and precision medicine (18–20). 3D organoid culture systems provide efficient preclinical cancer models of patient-derived organoids (PDOs), can better mimic the components of a tumor tissue, and can be efficiently established from patient specimens (18, 21, 22). The intratumor diversity in PDOs captures tumor heterogeneity at the single cell level and provides a valuable resource for cancer research. PDO cultures can be used to expanded over time while still retaining the mutational profiles of the parental tumors (23). On the contrary, traditional long-term 2D cultures have very high genomic instability. The highly conserved genomic landscape of PDOs is crucial to perform genotype-phenotype correlation analysis and to assess patient’s sensitivity to treatment. Although 2D culture is cheaper and relatively easy to maintain, the success rate of drug screening using 2D cultured tumor cells is very low, and the results are often conflicting. This may be because the 2D model cannot accurately reflect and maintain the tumor characteristics and complex cell-extracellular matrix interactions. Newly developed organoid culture platforms enable routine primary culture of resected human tumor tissues (24, 25).Numerous PDOs have been established from tissues derived from patients’ tumors, including colon (9), liver (25), gastric (26), lung (27), bladder (28), breast (29), and pancreatic cancers (30, 31) and head and neck squamous cell carcinoma (32). PDOs can be used to generate a well-annotated living cancer biobank as a resource for drug discovery and personalized therapy (33, 34). Although there are established PDOs generated from epithelial tissues, PDOs generated from non-epithelial cells are still rare. Sarcomas, malignant neoplasms originating from mesenchymal cells, have a high level of histopathological heterogeneity (35). Currently, several 3D sarcoma models with or without scaffold have been established from osteosarcoma, chondrosarcoma, Ewing sarcoma and soft tissue sarcoma (36–38). However, a standard protocol to generate sarcoma-derived organoid models has not yet been established. Therefore, we expect more advanced innovations to break through the bottleneck of developing sarcoma organoid culture and applications in the future, such as capturing the biological characteristics of native sarcomas in drug screening.

The tumor microenvironment (TME) includes vascular structures, extracellular matrix, and immune cell components, including lymphocytes, macrophages, myeloid-derived suppressor cells, dendritic cells, and natural killer cells (24). Cellular interactions in TME often determine drug response and the fate of the tumor. Functionally, the TME provides conditions for tumor progression and metastasis (39, 40). The recently developed PDO cultures provide an outstanding system to model patient-specific tumor-immune interactions. For instance, the co-culture of patient-derived cancer-associated fibroblasts and peripheral blood lymphocytes with pancreatic cancer organoids has been used to assess lymphocyte migration towards organoids in Matrigel and the activation status of myofibroblast-like cancer-associated fibroblasts (41). Co-culture of non-small-cell lung cancer and colorectal cancer organoids with autologous peripheral blood lymphocytes generates tumor-reactive T cells, and these T cells have the ability to kill tumor cells derived from the parental tumor tissue (20). In addition, culturing patient-derived organotypic tumor spheroids in microfluidic devices preserve endogenous immune cells, and this approach can model the tumor’s response to PD-1 blockade.

With the advancement of technology, many highly reproducible and controllable approaches have been developed to generate the microenvironment of human cancer bioengineered 3D organoid platforms that closely mimic *in vivo* tumor conditions (42). These platforms, such as organ-on-a-chip, can offer individual empirical data to better determine a patient’s drug response (43). Organ-on-a-chip is a multi-channel microfluidic cell culture device that includes multiple cell types to model the structure and function of the parental tissue (42, 44). Organoids develop from self-organizing stem cells to recapitulate the key physiological and pathological characteristics of their parental tissues. By integrating living human self-organizing organoids with organ-on-a-chip engineering, physiologically relevant microenvironments can be generated, and the resulting organoids-on-a-chip platform can combine the best features of both approaches to provide a model truly representing the complex characteristics of cancer progression (45). As a strategic integration, organoid-on-a-chip technology provides a superior *in vitro* platform for preclinical screening of chemotherapy drugs and predicting outcomes of radiotherapy and chemotherapy regimens.

**Figure 1:**
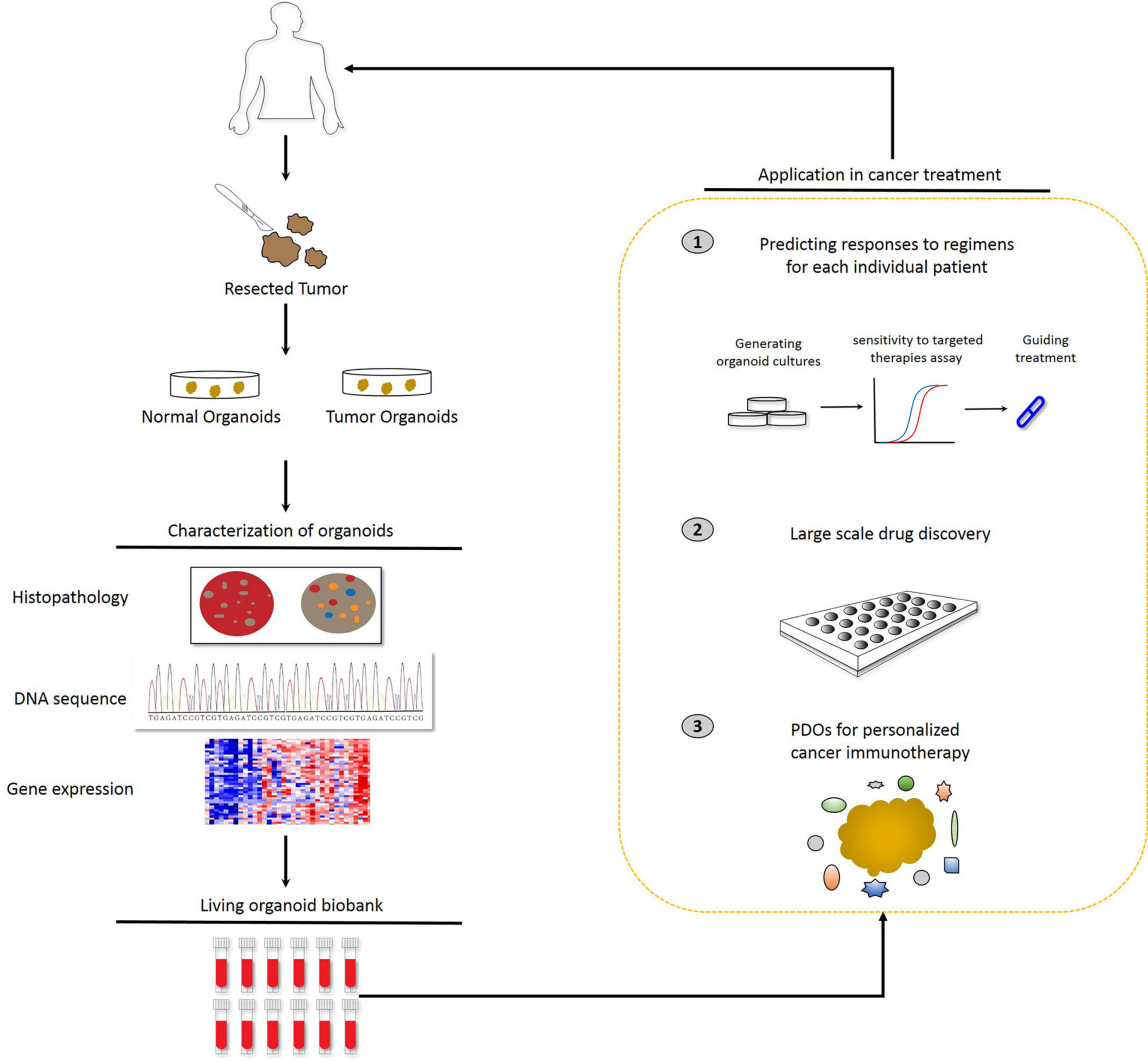
Potential applications of patient-derived organoids (PDOs). Identification of PDOs was performed *via* next-generation sequencing and comparison with the histology and pathology of the parental tumors. PDOs are suitable for drug sensitivity testing and drug selection to predict patient response and guide treatment at the individual level. In parallel, PDOs will be preserved as a living cell biobank and the organoid model is accessible for precision medicine.

In this review, we introduce the experimental approach of deriving organoids from adult stem cells, which can be generated directly from the epithelium of organs and explore how organoid cultures serve as a basis for developing a variety of microfluidic organ-on-a-chip platforms for clinical applications. In addition, we focus on patient-derived tumor organoids (PDTOs) in individualized cancer treatment and illustrate the advantages and limitations of PDTO biobanks as a resource for preclinical models and in enabling precision medicine **(** **Figure 1**).

## Frontier application of PDOs

### PDOs in Precision Medicine

Currently, patients with similar cancer types receive cognate treatments, but these treatments do not always achieve a uniform outcome across patient populations. Moreover, regardless of whether patients have undergone neoadjuvant chemoradiation or surgical treatment, individual drug response cannot be tested prior to treatment. In addition, recurring tumors may differ from the initial surgically resected tumors. Despite obvious interpatient heterogeneity, most clinical drugs are not developed using molecular biomarkers, except for some that target specific pathway mutations. To personalize cancer treatment, individual drug sensitivity assays with PDOs are progressively improving by recapitulating more physiological and pathological characteristics of tumors. Therefore, PDOs should be applied to drug screening and guide clinical treatment to improve prognosis. Traditionally, precision therapies have been performed by using mutational biomarkers; however, these biomarkers often lack a considerable tumor mass due to intratumor heterogeneity. As a result, treatments targeting these markers do not always elicit desirable patient responses. PDO models have been utilized in drug discovery (21) to explore the cytotoxicity of therapeutic candidates (46–49) and to enable personalized cancer treatments (18, 50). Recent studies on the generation and use of PDOs are summarized in **Table 1** (18, 21, 27–29, 33, 51–62). Using 19 colorectal cancer organoid lines, Van de Wetering et al. screened 83 drugs, including targeted inhibitors (18). Ooft et al. used PDOs to predict the response to chemotherapy in patients with metastatic colorectal cancer, and these results offer a chance to assess the reproducibility and applicability of organoid-based drug screening (63). Sachs et al. tested the response of six drugs targeting the human EGF receptor signaling pathway in 28 organoid lines and confirmed that breast cancer organoids serve as a superior physiologically relevant model for *in vitro* drug screening (29). Similarly, Yan et al. performed large-scale drug sensitivity screening using 37 anticancer compounds in nine gastric cancer organoids derived from seven patients (33). Vlachogiannis et al. applied patient-derived cancer organoids to predict the clinical outcomes of gastrointestinal cancer patients undergoing chemotherapy, targeted drug therapy and immunotherapy (64). By comparative analysis of the drug sensitivity of patients with metastatic gastrointestinal cancers and that of corresponding PDO models, they showed that the PDO model had a very high accuracy in predicting drug responses (64). Lee et al. screened 50 drugs in organoid models of bladder cancer, expressing the fibroblast growth factor (FGF) receptor, mitogen-activated protein kinase, and the mechanistic target of rapamycin inhibitors (28). Using 27 liver cancer organoid lines from five patients, Li et al. screened 129 cancer drugs and demonstrated that a subset of drugs induced a uniform toxic response across patient samples while the response to other drugs was heterogeneous (60). Pauli et al. performed a complete genomic analysis of four patients and high-throughput screening of 160 drugs using cancer organoids, and showed that 3D cultures are better than 2D cultures in identifying suitable individual or combination drugs for individual patients (21). These cancer organoids were derived from patients with metastatic and primary tumors, including prostate, bladder/ureter, kidney, colon/rectum, brain, pancreas, breast, stomach and esophagus, soft tissue, small intestine, lung, liver, adrenal gland, uterus, ovary, appendix and thyroid cancer. Brandenberg et al. reported an automated high-throughput screening system based on organoid cultures that could analyze thousands of individual gastrointestinal organoids within a polymer-hydrogel substrate (65). This 3D culture system significantly reduced the consumption of expansion reagents and was suitable for large-scale drug screening. Kim et al. reported an effective method for generating a living biobank of 80 lung cancer organoids (27). The drug responses of these organoid lines were consistent with interpatient and intratumor heterogeneity, indicating that cancer organoids are physiologically relevant drug screening platforms. Yao et al. established a living organoid biobank of locally advanced rectal cancer and showed that PDOs could predict chemoradiation responses in patients (66). Wang et al. reported a blinded study that found a PDTO model to be accurate in predicting chemotherapy responses in stage IV colorectal cancer (67).

**Table 1:** Application of drug screening with organoid culture platforms.

Cancer Type	Organoid Type	Library	Compounds Tested	Cases Tested	Assay Conditions	Refs
Bladder	CSC-derived	Target-known inhibitors + chemotherapy drugs	50	11	Matrigel	(28)
Breast	CSC-derived	EGFR/AKT/mTORC pathway inhibitors	6	28	BME	(29)
Breast	CSC-derived	CDK4/6 and BCL2 signaling pathway inhibitors	3	3	BME	(51)
Breast	CSC-derived	Docetaxel, Doxorubicin P4HA inhibitor	3	1	BME	(52)
Colorectal	CSC-derived	Target-known inhibitors + chemotherapy drugs	83	19	BME	(18)
Colorectal	CSC-derived	Target-know inhibitor + chemotherapy drugs	8	19	Matrigel	(53)
Colorectal	CTOS	Target-known inhibitors	71	1	W/O Matrix	(54)
Colorectal	CTOS organoids	Target-known inhibitors + FDA-approved drugs	2427	2	W/O Matrix	(55)
Endometrium	CTOS organoids	Target-known inhibitors	79	5	W/O Matrix	(56)
Endometrium	CSC-derived	Menin-MLL complex inhibitor	1/276	4	Matrigel	(57)
Gastric	CSC-derived	Approved anti-cancer drugs	37	7	Matirgel	(33)
Glioblastoma	CSC-derived	EGFR/PDGFR/Topoisomerase-II inhibitors and p53 pathway activator	4	3	Collagen–hyaluronic acid bioink	(58)
Glioblastoma	CSC-derived	Target-known inhibitors	64	2	Matrigel	(59)
Liver	CSC-derived	NCI-Approved Oncology Drugs Set VII	129	5	Matrigel	(60)
Liver	CSC-dervied	Target-know inhibitor + chemotherapy drugs	29	5	BME	(61)
Lung	CSC-derived	PARP /c-Met /EGFR inhibitor + Docetaxel	4	6	Matrigel	(27)
Ovarian	CSC-derived	Target-known inhibitors + chemotherapy drugs	22	10	Matrigel	(62)
Various	CSC-derived	chemotherapy drugs and targeted agents under clinical development	160 + 120	4	Matrigel	(21)

CSC, cancer stem cell; CTOS, cancer tissue-originated spheroid; BME, basement membrane extract. Chemo drugs; W/O, Water/Oil.

Monoclonal antibodies that target immune checkpoints, such as anti-CTLA4 and anti-PD-1, have been used to enhance anti-tumor T cell responses, increasing the overall survival rate in patients. Nigris et al. reported that PDOs were able to predict the patient’s response to PD-1/PD-L1 inhibitor therapy in primary chordoma (68). Jenkins et al. established a microfluidic culture of an organoid tumor spheroid platform to test the response of patient-derived tumors to immune checkpoint blockade treatment (69). Using a PDO/immune cell co-culture model, Zavros et al. demonstrated that rapamycin blocked the transcriptional regulation of PD-L1 by GLI1 and GLI2, and concluded that it is a valid model to assess immunosuppressive myeloid-derived suppressor cell function (70). These results show that gastric cancer organoids and immune cell co-culture systems can be used to predict patient response to immune checkpoint blockade and CAR-T cell infusion.

A search of ClinicalTrials.gov database from May 2015 to June 2021 revealed organoid-related clinical trials with the purpose of evaluating the probability of PDTO models to accurately predict patients’ responses or resistance to existing chemotherapeutic agents (**Table 2**). These clinical trials mainly focused on the individualized treatment of patients with various tumors and showed numerous advantages of using PDOs in precisely testing the corresponding patient’s sensitivity to chemotherapy and targeted therapy. In addition, an increasing number of PDO-based clinical trials in recent years suggests a trend towards an increasing reliance on PDOs for clinical decision making in personalized medicine. Nevertheless, clinical trials based on PDO models are still focused on tumors with relatively high morbidity and mortality, such as colorectal cancer, lung cancer, glioma, breast cancer, liver cancer, and pancreatic cancer. Moreover, PDOs are mainly derived from epithelial cells, and organoid culture techniques of non-epithelial cells are relatively immature and cannot be used in clinical trials.

**Table 2:** Summary of Clinical Trials of drug sensitivity with organoid methods.

Tissue Type	Source of Organoids	Aim of study	Estimated Enrollment	First Posted	Sponsors/Collaborators	ClinicalTrails.gov Identifier/Status
Astrocytoma	iPSC from patients’ peripheral blood mononuclear cell	To demonstrate that brain organoids can be used to test the impact of genetic mutants.	20	June 3, 2019	Sponsors and Collaborators: Assistance Publique Hopitaux De Marseille	NCT03971812/ Unknown
Breast cancer	breast cancer organ platform	Sensitivity Detection and Drug Resistance Mechanism (29 compounds)	300	April 24, 2019	Sponsor and Collaborators: Xijing Hospital, Xi’an, China	NCT03925233/ Enrolling by invitation
Breast cancer	Biopsy of primary or metastatic tumors	Drug Sensitivity Verification or Prediction (Paclitaxel)	50	June 1, 2018	Sponsors and Collaborators: Peking Union Medical College, Beijing, China	NCT03544047/ Unknown
Biliary Tract Cancer	Tumor resection	Multi-Platform Profiling with Organoid Drug Sensitivity Screening and ctDNA Monitoring	20	September 23, 2020	Sponsor: University of Washington Collaborators: Natera, Inc. SEngine Precision Medicine, Inc.	NCT04561453/ Recruiting
Colon Cancer	biopsy of RAS/RAF wild-type metastatic right colon cancer tumor lesion	Test the sensitivity and clinical consistency of cetuximab.	80	May 28, 2021	Sponsor: Danwang Medical Technology (Shanghai) Co., Ltd, China Collaborator: Fudan University, China	NCT04906733/ Recruiting
Cholangitis/Cholangiocarcinoma	Cholecystectomy (gallbladder removal); bile and biliary brushings	Characterization of Biliary Cell-derived Organoids	300	February 15, 2021	Sponsors and Collaborators: Mayo Clinic; National Institute of Diabetes and Digestive and Kidney Diseases (NIDDK)	NCT04753996/ Recruiting
Cystic Fibrosis	Rectal Biopsy and Suction biopsy or forceps biopsy (CF and R334W mutation)	Investigate the response to ivacaftor/tezacaftor in patients with CF and a R334W mutation.	30	February 5, 2020	Sponsor: Universitaire Ziekenhuizen Leuven; Collaborators: Vertex Pharmaceuticals Incorporated KU Leuven University of Lisbon	NCT04254705/ Not yet recruiting
Esophageal Cancer	Biopsy by diagnostic EUS	Prospective evaluation of chemoradioresistance	100	September 14, 2017	Sponsors and Collaborators: University Medical Center Groningen, Netherlands	NCT03283527/ Unknown
Familial adenomatous polyposis, Crohn and ulcerative colitis	intestinal biopsies (From Inflammatory Bowel Disease and Intestinal Polyposis Patients)	ISC and organoid characterization	120	August 22, 2016	Sponsors and Collaborators: University Hospital, Toulouse, France	NCT02874365/ Recruiting
Glioblastoma	Tumor biopsy ('left-over' tumor tissue)	Explore Resistance Mechanisms	60	April 30, 2021	Sponsor and Collaborators: Maastricht Radiation Oncology, Netherlands	NCT04868396/ Active, not recruiting
Glioma	Tumor resection and blood sampling	Establishing living biobank	50	April 29, 2021	Sponsor: Maastricht Radiation Oncology Collaborators: Maastricht University Medical Center Zuyderland Medisch Centrum Ziekenhuis Oost-Limburg	NCT04865315/ Active, not recruiting
Gut	Biopsy specimens (patients with and without hypertension who routinely undergo colonoscopy)	Determine if there are fundamental differences in the gut epithelium in hypertension compared to normotension.	50	August 4, 2020	Sponsor: University of Florida, United State Collaborator: National Heart, Lung, and Blood Institute (NHLBI)	NCT04497727/ Not yet recruiting
Human Gut Sensory Epithelial Cells	Endoscopic and colonoscopic biopsies	Study the biology of innervated sensory epithelial cells	50	September 5, 2016	Sponsor and Collaborators: Duke University	NCT02888587/ Recruiting
Head and Neck Cancer	Constitution of tumor and blood samples	Predicting the response to patients' treatments	98	February 7, 2020	Sponsors and Collaborators: Centre Francois Baclesse, France	NCT04261192/ Recruiting
Intestine	Small intestinal biopsies (A. healthy controls; B. patients with Food intolerances or Food allergy, patients with inflammatory bowel disease, irritable bowel disease, gluten sensitivity, short bowel syndrome)	The effect of nutrient antigens or therapeutic agents	375	August 22, 2017	Sponsors and Collaborators: University of Erlangen-Nürnberg Medical School, Germany	NCT03256266/ Recruiting
Kidney Cancer	Tumor resection, Blood and Urine sample	Establish a reliable and effective method to cultivate kidney cancer cells	20	April 13, 2020	Sponsors and Collaborators: Chinese University of Hong Kong	NCT04342286/ Recruiting
Lung Cancer	Surgical specimens	Establish long term culturing and bio-banking conditions, and Predict Treatment Response	30	April 26, 2021	Sponsors and Collaborators: Maastricht Radiation Oncology, Netherlands	NCT04859166/ Recruiting
Lung cancer	Resection of tumor tissue	Drug response testing	50	June 7, 2019	Sponsors and Collaborators: University Hospital, Geneva, Switzerland	NCT03979170/ Recruiting
Lung Neoplasm	Lung Tumor Resection and Circulating Tumor Cells	Creation a living biobank of PDOs from Stage I-IV lung cancer patients; Treatment Response of Organoids	150	August 31, 2018	Sponsors and Collaborators: The University of Texas Health Science Center at San Antonio, United States	NCT03655015/ Recruiting
Liver and Pancreatic Cancer	Tumor resection	Develop *in Vitro* Models of Liver, Biliary and Pancreatic Cancer	75	May 7, 2015	Sponsor: Cambridge University Hospitals NHS Foundation Trust Collaborators: The Gurdon Institute Ann McLaren Laboratory of Regenerative Medicine, UK	NCT02436564/ Unknown
Meningioma	Surgical specimens	Establishment and Characterization of Meningioma PDOs	30	July 21, 2020	Sponsors and Collaborators: Chinese University of Hong Kong	NCT04478877/ Recruiting
Multiple Myeloma	Marrow aspirates	Test chemosensitivity in relapsed multiple myeloma	70	March 26, 2019	Sponsor: Wake Forest University Health Sciences Collaborator: National Cancer Institute (NCI), United States	NCT03890614/ Recruiting
NSCLC	Surgical specimens and whole blood	High Throughput Screening Device Based on 3D Nano-matrices and 3D Tumors With Functional Vascularization	100	April 1, 2021	Sponsors and Collaborators: University Hospital, Strasbourg, France	NCT04826913/ Not yet recruiting
NSCLC	Resection tissue or biopsy tissue of NSCLC	Drug Sensitivity Correlation Between PDO Model and Clinical Response	100	March 5, 2018	K2 Oncology, Inc, China	NCT03453307/ Recruiting
NSCLC	Surgical specimens	Drug sensitivity test	100	March 5, 2018	Sponsors and Collaborators: K2 Oncology, Inc., China	NCT03453307/ Recruiting
Neuroendocrine neoplasm	Biopsy/surgical fresh tissue of gastroenteropancreatic neuroendocrine neoplasms and pancreatic ductal adenocarcinoma.	To use single-cell sequencing technology to explore neuroendocrine neoplasm molecular biological characteristics, tumor heterogeneity and cell subtypes.	200	June 16, 2021	Sponsors and Collaborators: Fudan University, China	NCT04927611/ Not yet recruiting
Ovarian Cancer	Operative specimens	Drug sensitivity (standard regimens: chemotherapies and targeted agents)	30	February 24, 2021	Sponsors and Collaborators: Chongqing University Cancer Hospital	NCT04768270/ Recruiting
Ovarian Cancer	Tumor biopsy	Drug response testing	48	September 18, 2020	Sponsors and Collaborators: Fondazione Policlinico Universitario Agostino Gemelli IRCCS, Italy	NCT04555473/ Recruiting
Pancreatic Cancer	EUS-FNA and EUS-FNB within the pancreatic cancer diagnostic process; Surgical specimens after neoadjuvant chemotherapy	Check for the reactivity to anti-cancer drugs used as neoadjuvant chemotherapy	300	March 2, 2021	Sponsors and Collaborators: Samsung Medical Center, Korea	NCT04777604/ Not yet recruiting NCT04736043/ Recruiting
Pancreatic Cancer	FNA and FNB	Evaluation and Comparison of the Growth Rate of Pancreatic Cancer Patient-derived Organoids to improve diagnostics and therapeutics	50	June 19, 2019	Sponsors and Collaborators: Technische Universität München	NCT03990675/ Recruiting
Pancreatic Cancer	EUS-FNA	Assess the responses of FDA-approved anti-cancer drugs	50	June 1, 2018	Sponsors and Collaborators: Ying Lv, China	NCT03544255/ Recruiting
Pancreatic adenocarcinoma	Biopsies of metastases or primary tumour tissue of pancreatic cancer	Establishing organoids	30	April 17, 2018	Sponsor: AMC-UvA Collaborator: Erasmus Medical Center	NCT03500068/ Recruiting
Prostate Cancer	Extended biopsy (metastatic prostate cancer)	Development of the organoid culture technique from metastases from patients with advanced form of prostate cancer	20	May 16, 2019	Sponsor: Centre Antoine Lacassagne, France Collaborator: Centre Meíditerraneíen de Meídecine Moleículaire UMR_S-1065	NCT03952793/ Recruiting
Rectal Cancer	Tumor biopsies	Establish a biospecimen collection protocol	20	May 1, 2020	Sponsors and Collaborators: Duke University	NCT04371198/ Recruiting
Rectal cancer	Pre-treatment biopsies	Predicting neoadjuvant chemoradiation sensitivity	80	July 5, 2018	Sponsors and Collaborators: Zhen Zhang, Fudan University, China	NCT03577808/ Unknown
Refractory Solid Tumours	Biopsy of HNSCC, Epithelial Ovarian, colorectal, breast cancer.	15-drug panel screening	35	May 29, 2019	Sponsors and Collaborators: National University Hospital, Singapore	NCT04279509/ Recruiting
Vaginal Cancer/Cervical Dysplasia/Cervical Cancer	Vaginal Biopsy	Primary Organoid Models for Anti-HPV Treatments	50	February 20, 2020	Sponsor: Centre Hospitalier Régional d'Orléans Collaborators: CNRS - Pr Chantal PICHON	NCT04278326/ Recruiting

NSCLC, Non-Small Cell Lung Cancer; HNSCC, Head and neck squamous cell carcinoma; PDOs, Patient-Derived Organoids; EUS, endoscopic ultrasound; AMC-UvA, Academisch Medisch Centrum - Universiteit van Amsterdam; UMCG, University Medical Center Groningen; EUS-FNA, EUS-guided fine-needle aspiration; EUS-FNB, EUS-guided fine-needle biopsy iPSC, Induced-Pluripotent Stem Cells; FAP, familial adenomatous polyposis.

Organoid culture can partially reveal interpatient heterogeneity in terms of sensitivity to anti-cancer drugs (71). Thus, it is critical to develop an organoid model system to predict drug sensitivity to estimate diversification in drug responses and reduce misguided selection of remedies in clinical trials. In addition, PDOs can be generated from various cancer patients and exhibit the intratumoral heterogeneity of the parental tumors. Herein, we have emphasized that organoid culture systems, especially PDOs, are suitable for precision medicine, including drug screening and prediction of individual patient’s response. As described above, colorectal cancer, breast cancer, gastric cancer, bladder cancer, liver cancer, and lung cancer organoids have been reported for drug screening and sensitivity. However, the application of conventional PDO models in precision medicine has numerous challenges. Although most tumor PDOs recapitulate the genetic composition of the parental tumor at early passages, the extent of genetic drift or the proportion of genetically stable cells in organoids at later passages has not been fully characterized (21). In addition, the lack of endogenous tumor-associated stromal components remains another key limitation of current organoid methods. Thus, the current PDO model is still unable to reflect all the characteristics of an organ. Although we have many urgent challenges to overcome, the continued development of PDOs incorporating immune and other stromal components may ultimately help actualize the promise of precision cancer therapies.

### Combination of PDOs and CRISPR/Cas9 Gene Editing

CRISPR/Cas9 genome editing in PDOs is used to establish transformation models, and eventually, for drug testing in the future. The use of CRISPR/Cas9 gene editing in PDOs has contributed to uncovering the functional basis of diverse oncogene mutations while also helping to correct the causing mutation in human cancers. Kuo et al. established the first human forward genetic modeling of a commonly mutated tumor suppressor gene, ARID1A, using CRISPR/Cas9 genome editing (72). Using this model, they obtained insights into early transformation mechanisms of ARID1A-deficient gastric cancers. Visvader et al. knocked out breast cancer-associated tumor suppressor genes using CRISPR/Cas9 editing to generate PDO model, and showed that the breast cancer organoid can be used for long-term growth (73). Meltzer et al. generated a novel PDO model to recapitulate aberrantly activated Wnt signaling by combining organoids and CRISPR/Cas9 genome editing (74). Using this model, they investigated the effect of an individual signaling alteration to human Barrett epithelial neoplastic transformation. Their research showed that the application of CRISPR/Cas9 genome editing creates an ideal Barrett epithelial PDO model to study ‘driver’ pathway alterations and improve our understanding of human tumorigenesis.

## Organoids-on-a-Chip and 3D Bioprinting

### Microfluidic Engineering Organoid Culture System

**Figure 2:**
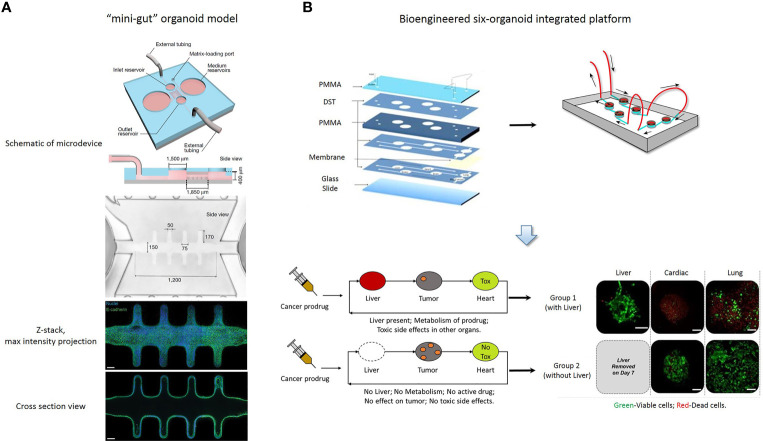
**(A)** A “mini-gut” organoid model is established in a microdevice containing 3D hydrogel. This microdevice guides self-organizing intestinal stem cells into functional organoids-on-a-chip. [Cited from (75)]. **(B)** A bioengineered six-organoid integrated platform is generated by microfluidically linked chambers, each containing liver, cardiac, lung, endothelial, testis, and brain organoids. Capecitabine treatment of a system containing liver, results in cytotoxicity in cardiac and lung organoids. Expectedly, this platform without liver organoids does not show significant toxicity. Green, Calcein AM-stained viable cells; Red, Ethidium homodimer-stained dead cells. PMMA, poly (methyl methacrylate); DST, double sided tape. Scale bars, 100 μm. [Adapted from (86)].

Recent studies of organoids have applied microfluidics and organ-on-a-chip technology in drug screening (75), in an attempt to overcome the shortcomings of organoid culture. Microfluidic cell culture technology has generated 3D culture devices that are now adapted to spheroid-based organotypic cultures and have been used to model organ microenvironments *in vitro* (76). This technology provides the possibility of precisely controlling the microscale to model physiological conditions and high-throughput approaches. Patient-derived organotypic tumor spheroids can be generated and evaluated within one to two weeks (69, 77, 78). Li et al. reported that the application of an air-liquid interface (ALI) provides sufficient oxygen supply to sustain organoid growth, which supports the generation of epithelial/mesenchymal hybrids without supplementation of exogenous growth factors (79, 80). The long-term 3D culture is a collagen-based ALI tumor organoid culture system that enables to expand the primary gastrointestinal cells as organoids for months (80). The ALI organoid method has been exploited to culture PDOs from normal and tumor specimens, including melanoma, renal cell carcinoma and non-small cell lung cancer (24). ALI PDOs preserves the heterogeneity of the parental tumor as well as the complex cellular network of the TME. Pavesi et al. developed a microfluidic device that could measure the changes in the antitumor efficacy of adoptive T cells in a 3D collagen microenvironment (81). Jung et al. devised a clinically relevant microphysiological microfluidic-based platform for drug sensitivity testing that could form tumor organoids with preserved morphological and genetic characteristics of the primary lung cancer (82). Torabi et al. designed micropatterned surfaces that integrated 3D cell culture with microfluidics through a hydrogel solution (83). Using the Cassie-Baxter mode, they created a diffusion and transfer pathway between the hydrogel and bulk fluid, providing an excellent option for PDO culture. The microfluidic 3D culture device could help PDOs retain the parenchyma and stroma, and enabled further assessment of new therapeutic modalities and elucidated the mechanism of chemotherapy resistance (24, 82, 84). Nikolaev et al. established a biomaterial microfluidic platform using tissue engineering and cell self-organizing approaches, which induced intestinal stem cells to establish a tube-shaped epithelium. Moreover, they demonstrated that this device could achieve a spatial arrangement similar to the crypt- and villus-like domains of the intestine *in vivo* (75). Interestingly, the mini-intestine specialized cell type, which is rarely found in conventional organoids and the luminal capability of the bioengineered system was sufficient to maintain long-term host-microorganism symbiosis (**Figure 2A**).

The combination of microfluidics and cell biology has led to the development of the organ-on-a-chip platform, which is a miniaturized biomimetic system that represents many physiological characteristics of living tissue, such as the 3D microarchitecture composed of multiple tissue types, dynamic mechanical and biomechanical forces, and functional multiple tissue integrations. Microfluidic organ-on-a-chip technology provides the possibility of easily controlling spatiotemporal flow thereby recreating a microenvironment for developing and maintaining the organoid model. Additionally, nutrient supply, shear stress and geometry can be easily controlled in an organ-on-a-chip platform, so that it is important to choose a critical function for this platform which can be achieved by designing a constructible simplified version of the real system. Achberger et al. presented a novel microphysiological model of the human retina, retina-on-a-chip, which included at least seven different essential retinal cell types derived from hiPSCs (85). The platform provided vasculature-like perfusion by microflow control technology and recapitulated the interaction of mature photoreceptor segments *in vitro*. In addition, they applied the anti-malaria drug chloroquine and the antibiotic gentamicin to reproduce retinopathic side effects and demonstrated the potential of retina-on-a-chip in drug development. Skardal et al. established a single and integrated multi-organoid body-on-a-chip system with a single recirculating perfusion system to maintain the viability and function of organoids derived from human tissue (86). These integrated systems could support six distinct tissue organoid types for at least 28 days, including the liver, cardiac, vascular, lung, testis, and either colon or brain. Interestingly, the six-organoid integrated platform was used to screen the toxicity of drug compounds at clinically relevant doses, and it was demonstrated that the functionality of one organoid influences the response of other organoids (**Figure 2B**). Kasendra et al. established a human duodenum intestine chip using organoids and organ-on-chips technology that mimicked intestinal tissue structure and functions and could be used for preclinical drug evaluation (87).

### 3D Bioprinting of PDOs

The construction of organoids still faces several challenges, including incorporation of vascular structures and immune system, precise architecture in space, and breakthrough in scale size. These vascular structures and immune systems can affect PDOs to predict the response of drug. The advantages of 3D bioprinting in biological reconstruction accelerates the process of organoid construction. Daly et al. developed a bioprinting approach to transfer spheroids into self-healing support hydrogels at high resolution, which achieves the precise manipulation of single spheroids and organoids (88). Ayan et al. discovered an “aspiration-assisted bioprinting” approach to improve the precise of biofabrication and bioprinted different biologics, including tissue spheroids, tissue strands, or single cells (89). In addition, Brassard et al. pursued an approach by printing organoid-forming stem cells to form centimeter-scale tissues that comprise self-organized features (90). The combination of 3D bioprinting and PDOs has successfully recapitulated part of the real structure and function of organoids, and achieved long-term expansion and improved drug testing. Kinsella et al. established bioprinting tumor models to maintain PDO sphere culture of gastric adenocarcinoma using hydrogels with alginate and gelatin (91). Bioprinted brain PDOs can be used for individual drug screening in neurological diseases. Using embedded 3D bioprinting and photocrosslinkable bioink, Shin et al. exploited a 3D brain-like co-culture construct that was composed of heterogenous neural populations with neurospheroids and glia (92). The study showed that the engineered brain organoid exhibited the capability to differentiate into neuronal cells, and the platform may be used to model neurological disease and drug discovery. The use of 3D bioprinting platforms to generate and culture organoids can improve reproducibility to a certain extent and promote the standardization of protocols. Although 3D bioprinting has been used in many organoid platforms, it still has numerous challenges, such as precise construction, printing speed, and suitable biomaterials. First, there is a gap in scale between organoids and actual organs: organoids are only up to a few cubic millimeters in size, which is a million times smaller than actual organs. Second, the long duration of the current manufacturing process may lead to hypoxia related damage by interrupting the continuous supply of nutrients and oxygen levels in the culture system. In addition, a single vasculature is insufficient for organoid development in the later stages of 3D printing organoid culture. Third, although bioprinting technology can effectively control the precise arrangement of cells, a precise construct is still difficult to achieve. Although challenges remain in the bioprinting organoid field, printable bioink and bioprinting strategies will be further developed in the future. Biomaterials, cell and matrix components of organoids, and the scale of organoids is the same as that of an organ. With breakthroughs in bioprinting organoid technologies and microfluidic culture systems, these challenges will be overcome and 3D organ bioprinting will eventually be realized.

## Organoid Biobanking and Ethical Concerns

### Living Organoid Biobanks

**Figure 3:**
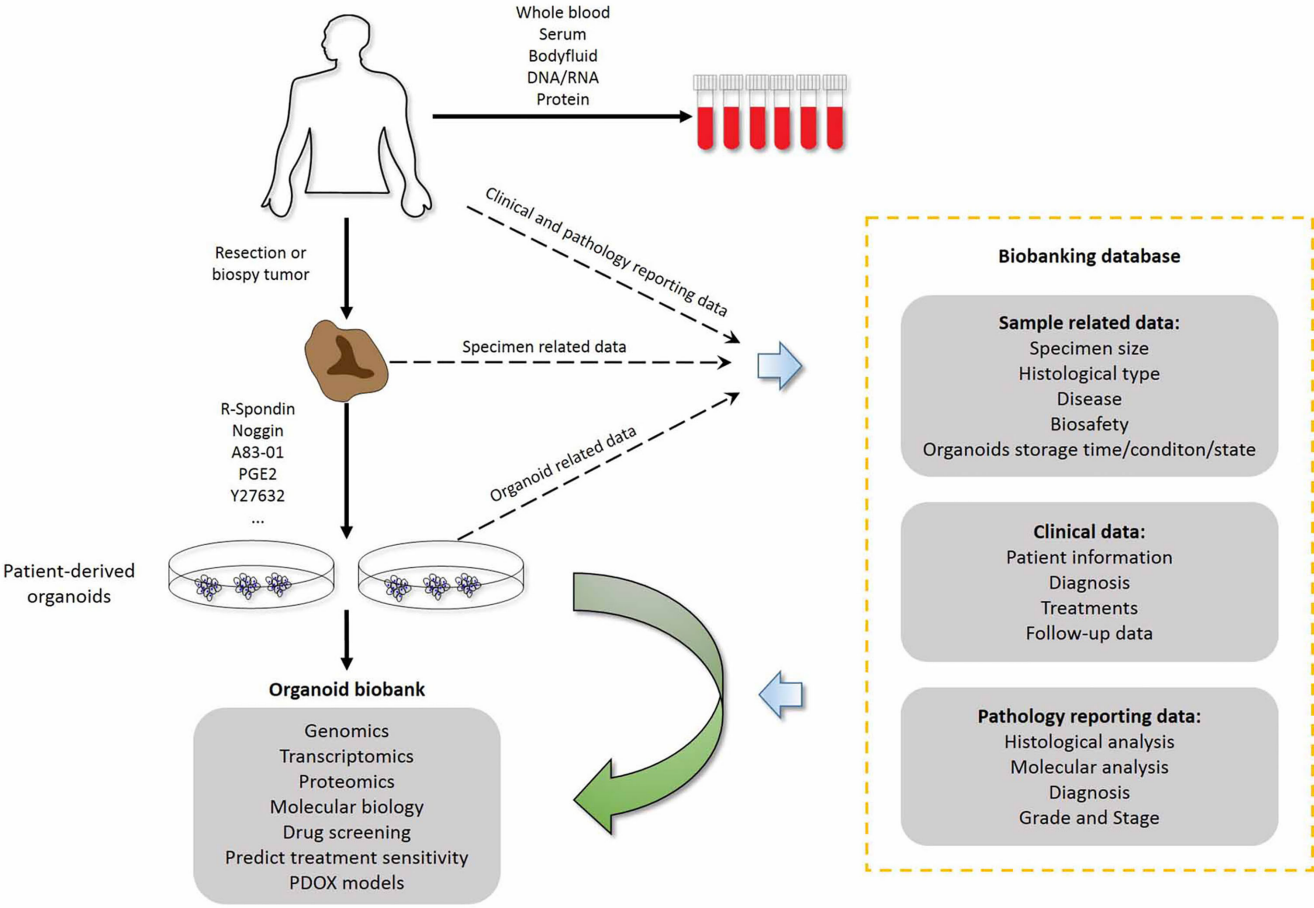
Combination of living organoid biobank and databases improves cancer research and precision medicine. Patient-related data are available through the hospital information system and contain sensitive patient information that external researchers cannot access. Researchers who have obtained ethics committee approval can collect sample-related anonymous information from the biobank data management system, and obtain the organoid model and fresh frozen tissue from the biobanking infrastructure. Therefore, researchers can use organoid models for drug screening and testing chemotherapy response at the individual patient level. PDOX models, Patient-derived organoid xenograft models.

For individualized cancer treatment, a bridge between clinical practice and translational research is urgently needed. Personalized therapies are based on the molecular and histopathological features of each patient’s tumor. In addition to traditional tissue and biomolecular-based biobanks, the establishment of a “living organism biobank” is receiving increasing attention, and one of its representatives is organoid biobanks. PDTOs can be passaged and cryopreserved, providing a chance to establish living biobanks with higher clinical relevance to the patients. PDO libraries allow in-depth investigation of tumor characteristics *in vitro*. Organoid biobanks, combined with drug sensitivity testing and next-generation sequencing, now support clinical decision-making and clinical trial performance analysis (**Figure 3**). Van de Wetering et al. first established a living colorectal cancer organoid biobank and described that the organoid culture platform can be exploited for genomic and functional research at the level of the individual patient (18). They provided detailed characterizations of a colorectal cancer biobank, including whole-exome sequencing, copy number analysis, histology and drug screening. Meanwhile, Geurts et al. described a cystic fibrosis intestinal organoid biobank, representing 664 patients (93). In addition, Fujii et al. generated a colorectal cancer organoid biobank that included 52 tumor subtypes and discovered that several organoids obtained new genetic mutations during passage, indicating that current research has not completely avoided the genetic instability of cancer organoids during long-term passage (94). These experimental results show the enormous potential of large-scale PDO biobanks that represent hereditary diseases. **Sachs** et al. established a living biobank of over 100 breast cancer organoid lines from a wide variety of primary and metastatic tumors (29). Moreover, they analyzed breast cancer organoids to characterize various profiles by large-scale sequencing and drug screening and generated a well-defined living biobank. These analyses ensured that the characteristics of breast cancer organoids were consistent with those of normal and tumor tissues from patients. These results indicated that PDO biobanks are more suitable for rare human cancer subtypes that are difficult to establish as immortalized cell lines. Yan et al. generated a gastric cancer organoid biobank derived from normal, dysplastic, cancer, and lymph node metastatic patients, and it retained different molecular subtypes (33). This biobank preserved features such paired tumor tissue germline DNA information, which is critical for future reference and prediction of patient responsiveness and sensitivity to anti-tumor treatments. Amieva et al. proposed a protocol to rapidly establish apical-out polarity and maintain the integrity and secretory function of epithelium (95). This protocol provides a tool for establishing a living gastrointestinal organoid biobank that can be used to study the impact of host-microbe interactions on epithelial function. Beato et al. established a living biobank of organoids from 15 patients with intraductal papillary mucinous neoplasms (IPMN) of the pancreas (96). These PDOs recapitulated the molecular and histopathological characteristics of the parental IPMN tumors, and the success rates for organoid generation from IPMN tumors and normal pancreatic tissues were similar to those of previous reports wherein the success rates were up to 80% and 87%, respectively (30, 31, 97–99). Jacob et al. reported the generation of patient-derived glioblastoma organoids that were suitable for constructing a biobank and modeling immunotherapy responses. With the complexity of cancer types dictates the outcome, the key advantage of these biobanks is that they provide cancer organoid cultures representing the complexity of different tumor subtypes. These cancer and normal organoids accurately reflect patient’s sensitivity to drugs and their tolerance to drug toxicity. An increasing number of cancer biobanks has been reported, but most of the existing organoid culture protocols are only suitable for epithelial carcinomas.

As we have described, only a few living tumor biobanks have been established by PDO technology, including colorectal cancer, breast cancer, gastric cancer and glioblastoma. In addition, fewer non-epithelial cancer-derived organoids have been established, such as glioblastoma (100) and childhood kidney cancers (34). Therefore, the generation of more organoid cultures from non-epithelial cancers should be promoted in the future. Based on the current status, more exploration should be performed to obtain living biobanks of rare tumor organoids. In addition, standardization of organoid production is needed to control the quality of PDOs, to improve the reproducibility and scalability, and to avoid the diversity of organoids. Based on the current research status, PDOs cannot fully recapitulate the natural characteristic of the parental tumor, which results in many uncertainties for the promotion of innovative clinical applications of living biobanks in the future.

### Ethical Concerns of PDO Research

Although advances in 3D models allow for more complex products to be generated from human tissues, the progress of human organoids may be hindered by ethical concerns. Vasiliki Mollaki analyzed several serious challenges posed by organoid use and biobanking and provided many unique and profound insights to promote the healthy development of organoid research and application (101). He provided an in-depth discussion on ethical challenges in organoid use, which includes the source of stem cells, informed consent of cell donors, issues specific to brain organoids and multi-organoid complexes, gene editing, creation of chimeras, organoid transplantation, commercialization of organoids, patentability of organoids, treatment costs, issues of equity, misuse and dual use of organoids, and organoid biobanking (101). His main suggestion is the four-step approach to help increase the biomedical and social benefits of organoids: the first is related to existing regulations and guidelines, the second is related to special regulatory provisions, the third is public engagement and the fourth is continuous monitoring of rapid advancements.

Organoid biobanking and issues specific to brain organoids are our main concerns. As mentioned above, living organoid biobanking provides an important source for promoting the development of translational research. PDOs have an inevitable connection with the donor’s body, identity, and privacy, among others, which involves human rights issues of the donor, and should differ from the tissues and organs directly derived from the human body. However, there are no binding principles or legal norms defining the rights and duties of donors and biobankers. Organoids are also a technology and a tool; hence, with the increasing commercialization of human organoid-related products, more and more ethical challenges have begun to emerge, especially in drug development, preclinical prediction of patient drug responses, and toxicology testing (102). The conventional frameworks are inapt to capture the practical and ethical complexity of human organoid products. Lensink et al. indicates that commercialization of PDO biobanks raise challenges associated with commercial involvement, trust, and ownership (103). By conducting 21 semi-structured qualitative interviews, they indicated that academia, clinical care, biobanks and industry stakeholders do not belong to distinct domains, and suggest that participants should be regarded as “partner” rather than passive tissue or service providers. These efforts are aimed at establishing an ecosystem that maintains a sound balance between ongoing cooperation and a feasible and sustainable research climate, while making governance more responsible and fair. In addition, living organoid samples can be stored for a long time after being collected and cultivated, even longer than the lifespan of the donor, and follow-up research often fails to provide informed consent. At the current stage of organoid biobanking, there are no standardized and individualized informed consents that can cover all the specific concerns of donors, such as personal values and beliefs. Therefore, opt-out options should be available to allow donors to object to certain uses. In any case, the consent procedure is the central tenet of organoid biobank management to ensure the implementation of the principle of a voluntary and well-informed donation (101).

The ethical issues of special living biobank samples, such as brain organoids, should also draw our attention. The human brain organoid system has already been applied to modeling neurological diseases, including microcephaly, macrocephaly, autism, Miller-Dieker syndrome, Rett syndrome, Sandhoff disease, prenatal drug exposure, ZIKA virus infection, and neurodegenerative diseases (104). Trujillo et al. developed human cortical organoids to model early human brain network development and achieved complex oscillatory waves (105). Reardon et al. discussed the sentient states of brain organoids, and pointed out that a conscious brain should display a much more complex, unpredictable electrical activity than an unconscious one, which responds in simple and regular patterns (106, 107). Although brain organoids do not have neurological functions, these miniature organs constitute neural entities of human origin. Currently, most scientists and ethicists agree that consciousness has not been created; however, with the continuous advance of technologies, brain organoids may be induced to develop consciousness, sensation, and cognition, thus possessing characteristics related to human morality. Therefore, ethical stakes are much more complex than those of other organs. Hyun et al. provided their opinions on the ethics of brain organoids (108). They indicated that brain organoids lack the sensory inputs and a complex network structure and, thus, declared that peoples’ concerns about the moral status might be excessive. At the current stage of development, the degree at which brain organoids exhibit human consciousness is difficult to determine, and neuroscientists have not reached a consensus on the definition and measurement of consciousness. However, if the brain organoids could feel pain, the principles of animal welfare would be imposed at least. In addition, the informed consent still should be modified to prepare for the day when brain organoids will be conscious, because the existing informed consent does not reflect all possible connections between the cell donor and brain organoids. Boers et al. proposed a “consent for governance” model that includes privacy by design, participant engagement, benefit sharing and ethical oversight, which contributes to responsible innovation and clinical translation (102). Overall, conventional bioethical frames are inept in addressing the practical and ethical complexities of PDOs. Therefore, it is essential to develop binding legal norms that overcome most of the ethical dilemmas in this exciting field.

## Discussion

Models of 3D tumor spheroids preserve cell-cell contact and cell-matrix interaction, present a more clinically relevant resistome and improve the success rate of drug screening (109). Studies have shown that the gene expression profiles of 3D cancer spheroids are different from 2D cultures, recapitulating various features in genes associated with proliferation, survival and drug sensitivity (110). Tumor spheroids embedded in ECM preserve most characteristics of cell biology associated with cell-matrix interrelations, including interaction with basement membranes and interstitial matrix (111, 112). Therefore, despite higher cost compared to 2D cell culture models, 3D tumor spheroids are popular for drug screening and response testing. Current 3D culture tumor models include organotypic multicellular spheroids (from tumor tissues), tumor-derived organoids (from dissociated tumor tissues) and multicellular tumor spheroids (from cancer cell lines) (113). Traditional spheroid culture models involve supplementation with B27, EGF, and FGFs. Organoid culture supplements depend on the type of tissue, and major supplements include the Wnt pathway agonist, RSPO1, nicotinamide, N-acetylcysteine, FGFs, noggin and molecule inhibitors (9, 66, 114–116). PDOs recapitulate the intercellular interactions and the characterizations of histology and enable long-term cultivation and stable passage (117). Therefore, PDOs mimic the genotype and phenotype of parental tumor and effectively retain patient-specific tumor heterogeneity, which make them superior to traditional spheroid models for drug screening. However, there are several disadvantages with PDO models that need to be overcome, including high cost and the potential effect of matrix on therapeutic responses. In addition, one main concern in cancer treatment is intra- and intertumoral heterogeneity (118), which can result in inaccurate decision-making and partial treatment benefits. Organoids derived from a portion of a tumor just match the genomic portrait of that particular tumor region, and may not represent the genome map of the entire tumor. Therefore, organoid assays of tumors *in vitro* should take the spatial tumor heterogeneity into consideration. In addition, owing to patient diversity and varying spheroid culture protocols, the outcome may vary by the laboratory. Culture protocols should be formulated that are specific and standardized for organoids derived from individual organs.

Although PDO models mimic some key aspects of human tumorigenesis, they cannot fully recapitulate the complicated structure of the TME. Tumorigenesis and drug resistance are not only driven by gene alterations in the tumor cells but are also affected by the components of the TME, such as blood vessels, neurons, fibroblasts and immune cells. First, immune system could be polarized to contribute to tumor development during progressive growth phase. Therefore, effort has been made to rejuvenate the anti-tumor immune response in organoid culture systems. Intestinal epithelial organoids have been co-cultured with lymphocytes and macrophages, and showed a significant dynamic movement and continued proliferation activity (75, 119).

As presented in the previous section, PDTOs can predict the response of cancer patients to chemotherapy. However, these studies have several limitations. First, owing to the lack of an integral microenvironment, organoid models cannot mimic immunotherapy and antiangiogenic therapy. Second major limitation of the current protocols for organoid culture is the inability to part with animal-derived Matrigel or collagens. These extracellular matrices contain undetermined extracellular components, which may unexpectedly modify biological cell behavior. Third, organ-on-a-chip organoids are suitable for studying the mechanism of tumor metastasis. However, multiorgan metastasis has not yet been achieved in organoid models. Additionally, current cancer organoid cultures do not replicate accurate mechanical control and physical manipulations that occur *in vivo*. Engineered extracellular matrix has been reported, which, however, still cannot meet the requirements of fully functional organoids (120).

Organoids are less expensive than mouse models, but they are relatively expensive compared to traditional cell line models. The time required to establish an organoid model is a few weeks which is less than that in animal models but is still longer than in cell line models. High-throughput assays are required to decrease the time and cost of organoid generation as well as the input material needed to establish the culture. In this regard, microfluidic 3D culture has generated spheroid-based organotypic culture devices. Organoid-on-a-chip is also a microfabricated microfluidic culture platform that combines extracellular matrix and microstructures to simulate one part of the cytoarchitecture and tissue function (42). However, the microfluidic system cannot replicate the interactions between the tumor and the immune network that occurs *in situ* and is required for an accurate prediction of immunotherapy response *ex vivo*. Moreover, although intestinal organoid fragments on hydrogel have been applied to manufacture organoid arrays (65, 121, 122), they are not adequate to provide fully automated organoid culture for high-throughput assays. Finally, the generation of organoids and other human tissue products leads to ethical challenges, including gift versus market systems especially during the commercialized exchange of organoids, and the awakening of consciousness in brain organoids.

## Conclusion

Despite the remaining challenges, PDOs have a higher physiological and pathological relevance than traditional models, and human cancer organoid assays have great potential in guiding personalized therapies. Meanwhile, PDTOs allow to reliably preserve the molecular, cellular, and histopathological phenotypes of parental tumors and retain patient-specific tumor heterogeneity. Furthermore, organ-on-a-chip has been applied to organoids to accomplish physiological or pathological model systems that are closer to the state of the tissue *in vivo*. Future advancements in organoid technologies are anticipated to achieve a comprehensive cancer model system that recapitulates physiological conditions by integrating tumor parenchyma cells, vascular and immune cellular networks, and non-cellular TME. This robust model will provide a powerful tool for biomarker research, drug screening, and a more accurate prediction of therapeutic efficacy and eventually improve human health.

## Author Contributions

The concept of the manuscript was originated by ZZ and XC. The original manuscript was written by ZZ, with additions to the manuscript provided by LC. All authors contributed to the article and approved the submitted version.

## Funding

This study was supported by the Science and Technology Development Project of Jilin Province (20210402030GH, 20200601010JC, 2021C017, 20210204150YY).

## Conflict of Interest

The authors declare that the research was conducted in the absence of any commercial or financial relationships that could be construed as a potential conflict of interest.

## Publisher’s Note

All claims expressed in this article are solely those of the authors and do not necessarily represent those of their affiliated organizations, or those of the publisher, the editors and the reviewers. Any product that may be evaluated in this article, or claim that may be made by its manufacturer, is not guaranteed or endorsed by the publisher.
